# Molecular Mechanisms of Neutrophil Extracellular Traps in Promoting Gastric Cancer Epithelial–Mesenchymal Transition Through SERPINE‐1 Expression

**DOI:** 10.1002/jbt.70157

**Published:** 2025-03-10

**Authors:** Zhen Ma, Xiaolin Li, Shifeng Yang, Hao Yang, Ange Zhang, Nana Li, Xiaoming Zou

**Affiliations:** ^1^ Department of General Surgery The Second Affiliated Hospital of Harbin Medical University Harbin City China

**Keywords:** epithelial–mesenchymal transition, gastric cancer, neutrophil extracellular traps, SERPINE‐1

## Abstract

Gastric cancer remains a significant global health concern, with its progression and metastasis often associated with epithelial–mesenchymal transition (EMT). This study investigated the role of neutrophil extracellular traps (NETs) in promoting gastric cancer EMT by regulating SERPINE‐1 expression, which encodes plasminogen activator inhibitor‐1 (PAI‐1). Western blot and immunohistochemistry were used to detect protein expression. Cell Counting Kit‐8 was tested for cell proliferation ability using clones. The SERPINE‐1 gene was knocked down using lentivirus. Immunofluorescence was used to detect the co‐expression of proteins, and a Transwell assay and wound‐healing assay were used to investigate the migration ability of cells. Experimental conclusions were verified in vivo using a nude mouse model. We first demonstrated overexpression of PAI‐1 in gastric cancer tissues and cell lines. Subsequently, we found that NETs significantly enhanced the expression of EMT‐related markers. These changes were accompanied by increases in cell invasion, migration, proliferation and tumour sphere formation. To further elucidate the mechanism, we employed lentivirus‐mediated SERPINE‐1 knockdown to reverse NET‐induced EMT phenotype effectively. Mechanistically, we found that NETs activated the transforming growth factor (TGF)‐β signalling pathway via PAI‐1 as evidenced by increased expression of TGF‐β1, TGF‐βR1, TGF‐βR2, phosphorylated Smad2/3 and Smad4. Finally, in vivo experiments using a nude mouse model of gastric cancer liver metastasis confirmed that NET‐treated HGC‐27 cells exhibited enhanced metastatic potential and SERPINE‐1 knockdown abrogated metastatic potential. Our findings reveal a novel mechanism by which NETs promote EMT and metastasis in gastric cancer via the PAI‐1–TGF‐β axis. PAI‐1 can be used as a potential target for the treatment of gastric cancer, and the expression of PAI‐1 is closely related to the prognosis of patients with gastric cancer. Therapeutic strategies targeting NETs or PAI‐1 may help prevent EMT and metastasis of gastric cancer and improve clinical outcomes in patients.

## Graphical abstract

In this study, it revealed that neutrophil extracellular traps (NETs) promoted metastasis, proliferation and epithelial–mesenchymal transition (EMT) of gastric cancer cells. Mechanistically, NETs activated TGFβ signalling by inducing expression of SERPINE‐1 (PAI‐1) whose level in gastric cancer tissues and cell lines is upregulated. The activated TGFβ axis showed an increase of phosphorylated Smad2/3, and expression of Smad4, which were translocated into the nucleus, resulting in EMT phenotype. The EMT and metastasis of HGC‐27 and MKN‐45 in vitro and in vivo were inhibited through the knockdown of SERPINE‐1 by shRNA, providing new insights into potential therapeutic strategies for gastric cancer.

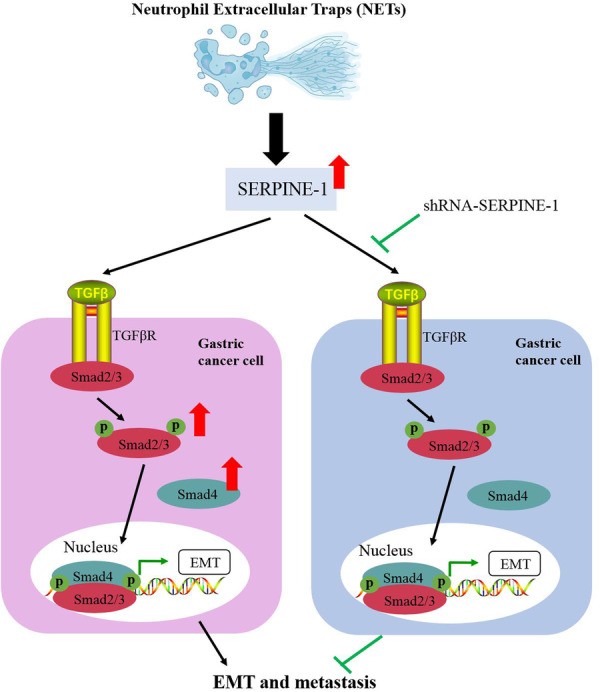

## 1 Introduction

Gastric cancer remains one of the leading causes of cancer‐related deaths worldwide, with its aggressive nature and propensity for metastasis contributing significantly to poor patient outcomes [1]. The epithelial–mesenchymal transition (EMT) is a critical process in cancer progression, enabling epithelial cells to acquire mesenchymal traits, thus facilitating invasion and metastasis [2]. While various factors have been implicated in promoting EMT in gastric cancer, the role of the tumour microenvironment, particularly inflammatory components, remains an area of intense research [3].

Neutrophil extracellular traps (NETs) are web‐like structures composed of extracellular deoxyribonucleic acid (DNA), histones and granular proteins released by activated neutrophils [4]. Initially described as a defence mechanism against pathogens, NETs have recently been implicated in cancer progression [5]. A recent study reported a NET‐related model for prostate cancer that has excellent predictive performance for predicting biochemical recurrences as well as chemotherapy efficacy [6]. In ovarian cancer, upregulation of RAC2 is associated with the formation of NETs and poor prognosis of ovarian cancer, and NET‐associated markers are reliable for ovarian cancer prognosis prediction and treatment evaluation [7]. Other studies have reported that NETs are related to many cancers, such as lung cancer [8], hepatocellular carcinoma [9] and colorectal cancer [10]. However, their specific role in gastric cancer EMT and the underlying molecular mechanisms remain poorly understood.

Plasminogen activator inhibitor‐1 (PAI‐1), encoded by the SERPINE‐1 gene, is a key regulator of the plasminogen activation system and has been associated with poor prognosis in various cancers, including gastric cancer [11]. Recent studies have suggested a link between PAI‐1 and EMT in cancer progression, but its relationship with NETs in the context of gastric cancer has not been explored [12].

This study aims to elucidate the molecular mechanisms by which NETs influence gastric cancer EMT through the regulation of SERPINE‐1 expression. We hypothesise that NETs promote gastric cancer EMT by upregulating PAI‐1, potentially through the activation of the transforming growth factor (TGF)‐β signalling pathway, a well‐known inducer of EMT [13]. We find a novel mechanism by which NETs promote EMT and metastasis in gastric cancer through PAI‐1 upregulation and subsequent activation of the TGF‐β signalling pathway. Our findings provide novel insights into the complex interplay between inflammatory components of the tumour microenvironment and cancer progression, potentially opening new avenues for therapeutic interventions in gastric cancer.

## 2 Methods

### 2.1 Cell Culture and Neutrophil Extracellular Trap Preparation

Human gastric cancer cell lines (GES‐1, HGC‐27, MKN‐45, KOTA III and AGS) were obtained from the American Type Culture Collection. Cells were cultured in RPMI‐1640 medium supplemented with 10% foetal bovine serum (FBS) and 1% penicillin–streptomycin at 37°C in a 5% CO_2_ incubator.

NETs were isolated from human neutrophils by following a previously described protocol [14]. Briefly, neutrophils were isolated from healthy donors' blood using density gradient centrifugation. Isolated neutrophils were stimulated with 100 nM phorbol 12‐myristate 13‐acetate for 4 h to induce NET formation. The NETs were collected, quantified by DNA content and either used directly or treated with DNase‐1 (100 U/mL) for 30 min at 37°C before use in experiments.

### 2.2 Immunohistochemistry

Gastric cancer tissue samples and adjacent normal tissues were obtained from patients undergoing surgical resection, with informed consent and approval from the institutional ethics committee. Tissue sections (4 μm) were deparaffinised, rehydrated and subjected to antigen retrieval using citrate buffer (pH 6.0). Endogenous peroxidase activity was blocked with 3% H_2_O_2_. Sections were then incubated with anti‐PAI‐1 antibody (1:200, Abcam) overnight at 4°C, followed by incubation with HRP‐conjugated secondary antibody; 3,3′‐diaminobenzidine was used as the chromogen, and sections were counterstained with haematoxylin.

### 2.3 Western Blot Analysis

Cells were lysed in a radioimmunoprecipitation assay buffer containing protease and phosphatase inhibitors. Protein concentration was determined using the bicinchoninic acid assay. Equal amounts of protein (30 μg) were separated by 10% sodium dodecyl sulfate–polyacrylamide gel electrophoresis and transferred to polyvinylidene fluoride membranes. The membranes were blocked with 5% non‐fat milk and then incubated with primary antibodies against PAI‐1, Vimentin, N‐cadherin, E‐cadherin, TGF‐β1, TGF‐βR1, TGF‐βR2, phospho‐Smad2/3, Smad4 and total Smad2/3 (all 1:1,000, Cell Signaling Technology) overnight at 4°C. After washing, the membranes were incubated with horseradish peroxidase‐conjugated secondary antibodies. Protein bands were visualised using enhanced chemiluminescence reagent and quantified by densitometry.

### 2.4 Transwell Invasion Assay

Matrigel‐coated Transwell chambers (8 μm pore size, Corning) were used to assess cell invasion. Cells (1 × 10^5^) were seeded in the upper chamber in serum‐free medium, with or without NETs (50 μg/mL) or DNase‐1‐treated NETs. The lower chamber contained a medium with 10% FBS as a chemoattractant. After 24 h, non‐invaded cells were removed, and invaded cells were fixed with 4% paraformaldehyde, stained with 0.1% crystal violet and counted under a microscope in five random fields.

### 2.5 Wound Healing Assay

Cells were grown to confluence in 6‐well plates, and a scratch was made using a sterile 200 μL pipette tip. After washing with phosphate‐buffered saline (PBS), the cells were incubated in a serum‐free medium with or without NETs (50 μg/mL) or DNase‐1‐treated NETs. Wound closure was monitored at 0 and 24 h using phase‐contrast microscopy. The wound area was measured using ImageJ software, and the percentage of wound closure was calculated.

### 2.6 Immunofluorescence

Cells grown on coverslips were fixed with 4% paraformaldehyde, permeabilised with 0.1% Triton X‐100 and blocked with 5% bovine serum albumin. Primary antibodies against PAI‐1, Vimentin, N‐cadherin and E‐cadherin (all 1:200, Cell Signaling Technology) were applied overnight at 4°C, followed by fluorescent‐conjugated secondary antibodies (1:500, Invitrogen) for 1 h at room temperature. Nuclei were counterstained with 4′,6‐diamidino‐2‐phenylindole. Images were captured using a Zeiss LSM 800 confocal microscope.

### 2.7 Sphere Formation Assay

Cells (1 × 10^3^) were seeded in ultra‐low attachment 96‐well plates in serum‐free Dulbecco's modified eagle medium/nutrient mixture F‐12 medium supplemented with B27, 20 ng/mL epidermal growth factor and 10 ng/mL basic fibroblast growth factor. NETs (50 μg/mL) or DNase‐1‐treated NETs were added where indicated. Sphere formation was assessed after 7 days using an inverted microscope. Spheres > 50 μm in diameter were counted.

### 2.8 Cell Proliferation Assay

Cell proliferation was measured using the Cell Counting Kit‐8 (CCK‐8) assay kit (Dojindo) according to the manufacturer's instructions. Cells (5 × 10^3^/well) were seeded in 96‐well plates and treated with NETs or DNase‐1‐treated NETs. At 24, 48, and 72 h, CCK‐8 reagent was added, and absorbance was measured at 450 nm using a microplate reader.

### 2.9 Lentiviral Short Hairpin Ribonucleic Acid Knockdown

Lentiviral vectors expressing short hairpin ribonucleic acid (shRNA)‐targeting SERPINE‐1 (shSERPINE‐1) or a non‐targeting control (shNC) were constructed and packaged in HEK293T cells using a third‐generation lentiviral system. Gastric cancer cells were transduced with the lentiviral particles at a multiplicity of infection of 10 in the presence of 8 μg/mL polybrene. Transduced cells were selected with 2 μg/mL puromycin for 7 days to establish stable knockdown cell lines.

### 2.10 Animal Studies

All animal experiments were approved by the Institutional Animal Care and Use Committee. Female BALB/c nude mice (6–8 weeks old, *n* = 5/group) were used, and HGC‐27 cells (1 × 10^6^; control, NET + shNC or NET + shSERPINE‐1) suspended in 50 μL PBS were injected into the spleen. After 2 weeks, the mice were sacrificed, and livers were harvested. Liver metastases were counted and measured using a dissecting microscope.

### 2.11 Statistical Analysis

Data are presented as mean ± standard error of the mean from at least three independent experiments. Statistical significance was determined using Student's *t*‐test for two‐group comparisons or one‐way analysis of variance with Tukey's post‐hoc test for multiple‐group comparisons. A *p* < 0.05 was considered statistically significant. All statistical analyses were performed using GraphPad Prism 8.0 software.

## 3 Results

### 3.1 Plasminogen Activator Inhibitor‐1 Is Overexpressed in Gastric Cancer Tissues and Cell Lines

**Figure 1:**
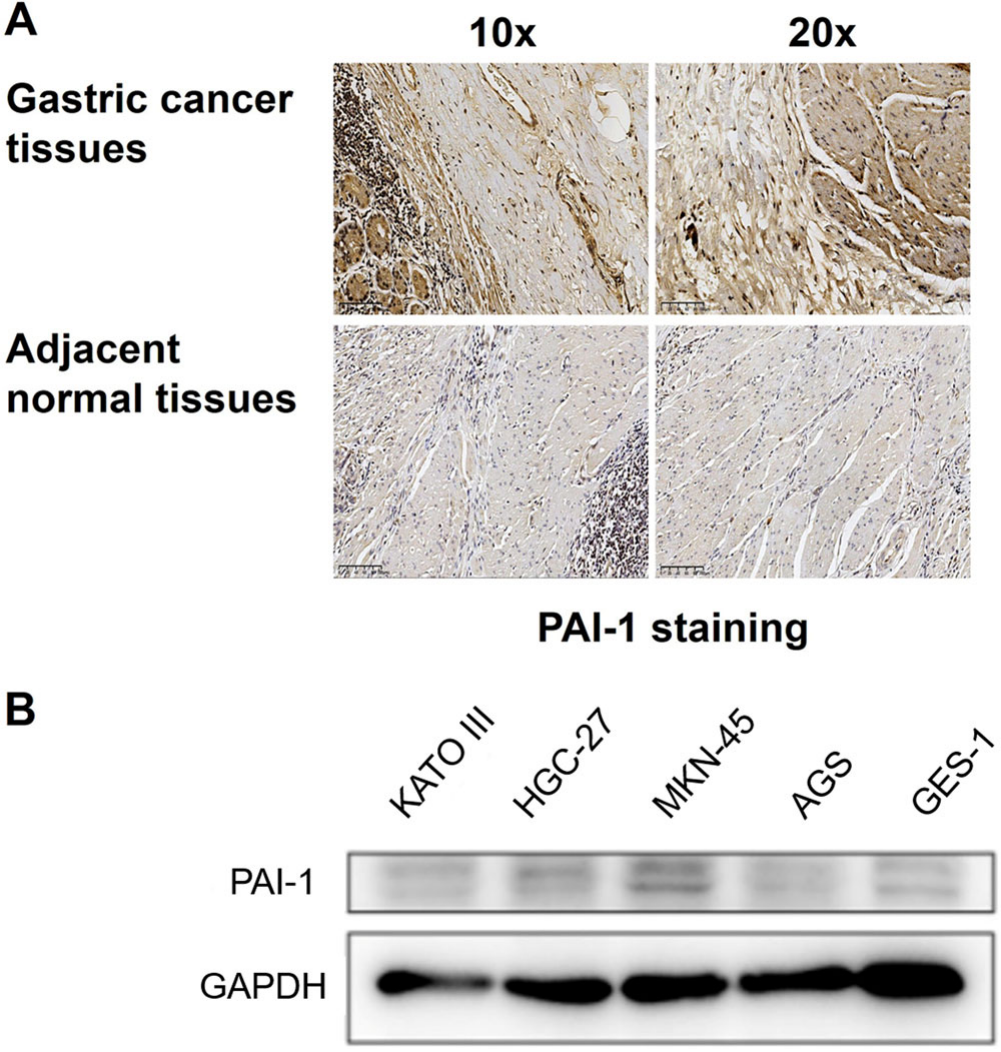
PAI‐1 is overexpressed in gastric cancer tissues and cell lines. (A) Representative immunohistochemical staining of PAI‐1 in gastric cancer tissues and adjacent normal tissues. Scale bar: 100 μm. (B) Western blot analysis of PAI‐1 expression in gastric cancer cell lines (GES‐1, HGC‐27, MKN‐45, KOTA III and AGS) compared to normal gastric epithelial cell line GES‐1.

PAI‐1 expression in gastric cancer correlates with tumour aggressiveness and poor prognosis [11]. To investigate the potential role of PAI‐1 in gastric cancer progression, its expression in gastric cancer tissues and cell lines was first examined. Immunohistochemical staining revealed strong PAI‐1 expression in gastric cancer tissues compared with adjacent normal tissues (Figure 1A). This overexpression was further confirmed at the protein level by western blot analysis of various gastric cancer cell lines (GES‐1, HGC‐27, MKN‐45, KOTA III and AGS) compared with the normal gastric epithelial cell line GES‐1 (Figure 1B). Notably, HGC‐27 and MKN‐45 cell lines exhibited the highest PAI‐1 expression levels and were therefore selected for subsequent experiments.

### 3.2 Neutrophil Extracellular Traps Promote Epithelial–Mesenchymal Transition in Gastric Cancer Cells

**Figure 2:**
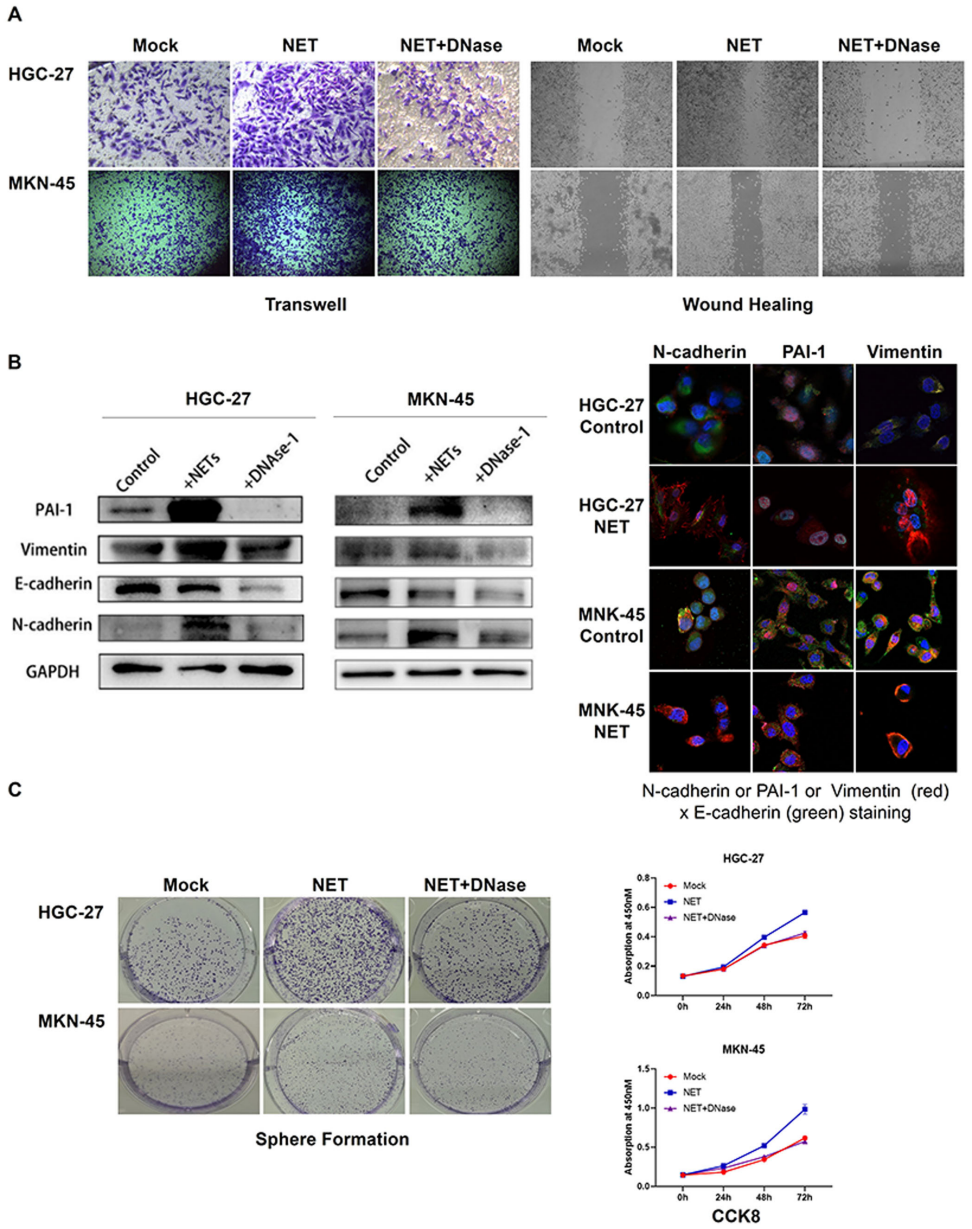
NETs promote EMT in gastric cancer cells. (A) Transwell invasion assay and wound healing assay of HGC‐27 and MKN‐45 cells treated with NETs or DNase‐1‐treated NETs. Representative images at 48 h were shown. (B) Western blot analysis of EMT markers (PAI‐1, Vimentin, N‐cadherin, E‐cadherin) in HGC‐27 and MKN‐45 cells treated with NETs or DNase‐1‐treated NETs. Additionally, immunofluorescence co‐staining of PAI‐1 and EMT typical markers (Vimentin, N‐cadherin, and E‐cadherin) in HGC‐27 and MKN‐45 cells treated with NETs. Scale bar: 20 μm. (C) Sphere formation assay and CCK‐8 proliferation assay of HGC‐27 and MKN‐45 cells treated with NETs or DNase‐1‐treated NETs. Representative images of sphere formation assay at 48 h were shown.

To elucidate the effect of NETs on gastric cancer EMT, HGC‐27 and MKN‐45 cells were treated with isolated NETs or DNase‐1‐treated NETs. Functional assays further supported the pro‐EMT effects of NETs. Transwell invasion assays showed enhanced invasive capacity of gastric cancer cells treated with NETs, which was reversed by DNase‐1 treatment (Figure 2A). Similarly, wound healing assays demonstrated increased migration of NET‐treated cells compared with the control or DNase‐1‐treated NET groups (Figure 2A).

Western blot analysis revealed that NET treatment significantly increased the expression of mesenchymal markers (Vimentin and N‐cadherin) and PAI‐1 while decreasing the epithelial marker E‐cadherin. Immunofluorescence staining corroborated these findings, showing increased expression of PAI‐1, Vimentin and N‐cadherin, with decreased E‐cadherin expression in NET‐treated cells. These changes were attenuated when cells were treated with DNase‐1‐digested NETs, suggesting that the integrity of the NET structure is crucial for its EMT‐promoting effects (Figure 2B).

To assess the impact of NETs on cancer stem cell‐like properties, sphere formation assays were performed. The NET‐treated cells formed significantly larger and more numerous spheres compared with control cells or those treated with DNase‐1‐digested NETs. Additionally, CCK‐8 proliferation assays revealed enhanced proliferation in NET‐treated cells, an effect that was abolished by DNase‐1 treatment (Figure 2C).

### 3.3 SERPINE‐1 Knockdown Reverses Neutrophil Extracellular Trap‐Induced Epithelial–Mesenchymal Transition in Gastric Cancer Cells

**Figure 3:**
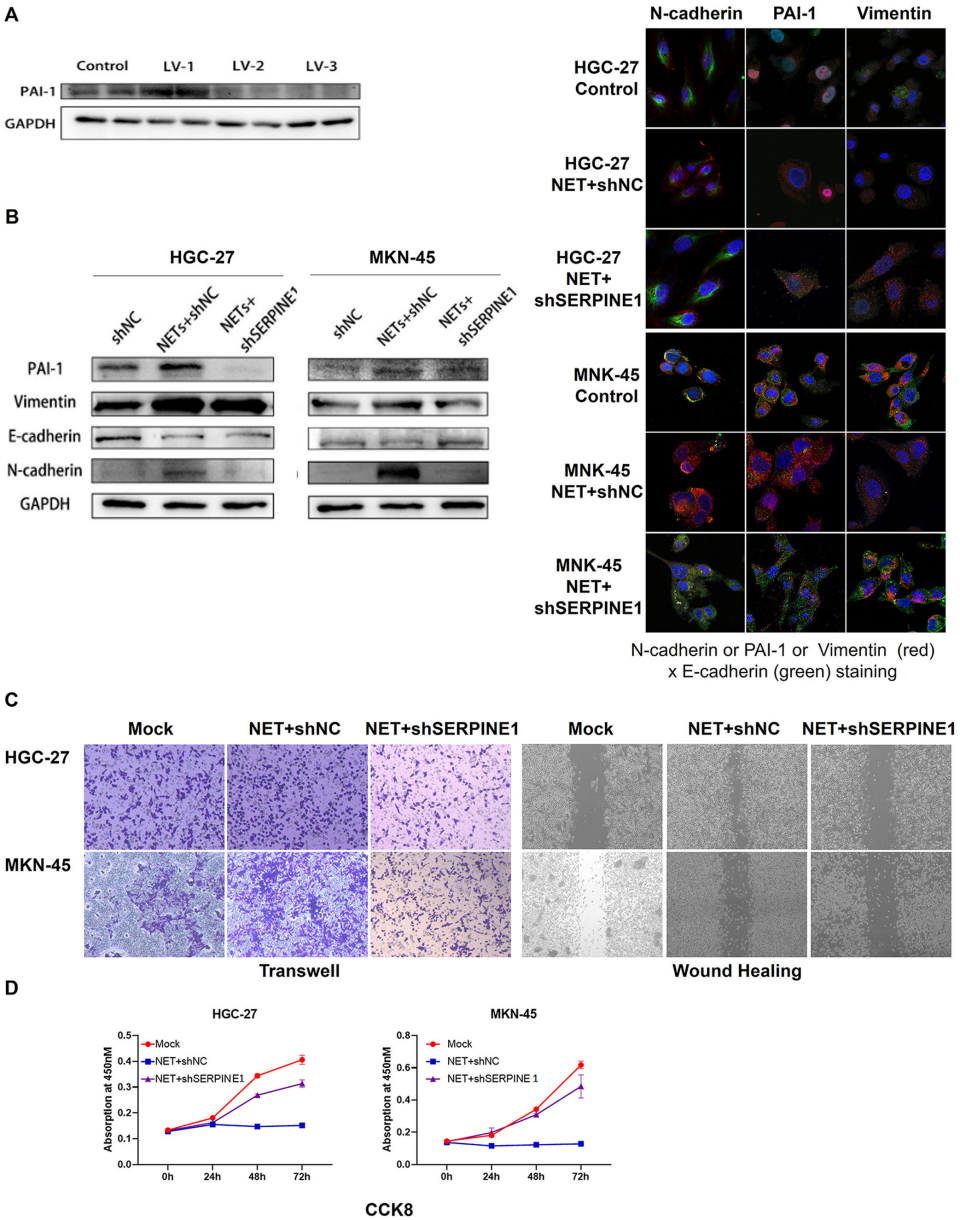
SERPINE‐1 knockdown reverses NET‐induced EMT in gastric cancer cells. (A) Western blot analysis confirming SERPINE‐1 knockdown efficiency in HGC‐27 and MKN‐45 cells. (B) Western blot analysis of EMT markers in control and SERPINE‐1 knockdown cells treated with NETs. Additionally, immunofluorescence co‐staining of PAI‐1 and EMT typical markers (Vimentin, N‐cadherin, and E‐cadherin) in control and SERPINE‐1 knockdown cells treated with NETs. Scale bar: 20 μm. (C) Transwell invasion assay and wound healing assay of control and SERPINE‐1 knockdown cells treated with NETs. Representative images at 48 h were shown. (D) CCK‐8 proliferation assay of control and SERPINE‐1 knockdown cells treated with NETs.

To investigate the specific role of PAI‐1 in NET‐induced EMT, stable SERPINE‐1 knockdown cell lines were established using lentiviral shRNA. The efficiency of knockdown was confirmed by western blot analysis, showing significantly reduced PAI‐1 protein levels in shSERPINE‐1 cells compared with shNC cells (Figure 3A).

The effect of SERPINE‐1 knockdown on NET‐induced EMT was then examined. Western blot analysis showed that SERPINE‐1 knockdown significantly attenuated the NET‐induced increase in Vimentin and N‐cadherin expression while partially restoring E‐cadherin levels. Immunofluorescence staining further confirmed these results (Figure 3B).

Functionally, SERPINE‐1 knockdown markedly reduced the NET‐enhanced invasive capacity (Figure 3C), migratory ability (Figure 3C) and proliferation rate (Figure 3D) of gastric cancer cells. These results strongly suggest that PAI‐1 plays a crucial role in mediating the pro‐EMT effects of NETs on gastric cancer cells.

### 3.4 Neutrophil Extracellular Traps Activate the Transforming Growth Factor‐β Signalling Pathway via Plasminogen Activator Inhibitor‐1

To elucidate the molecular mechanism underlying NET‐induced, PAI‐1‐mediated EMT, the TGF‐β signalling pathway—a well‐known inducer of EM—was examined. Analysis of the TCGA database indicated a potential link between PAI‐1 and the TGF‐β pathway in gastric cancer.

**Figure 4:**
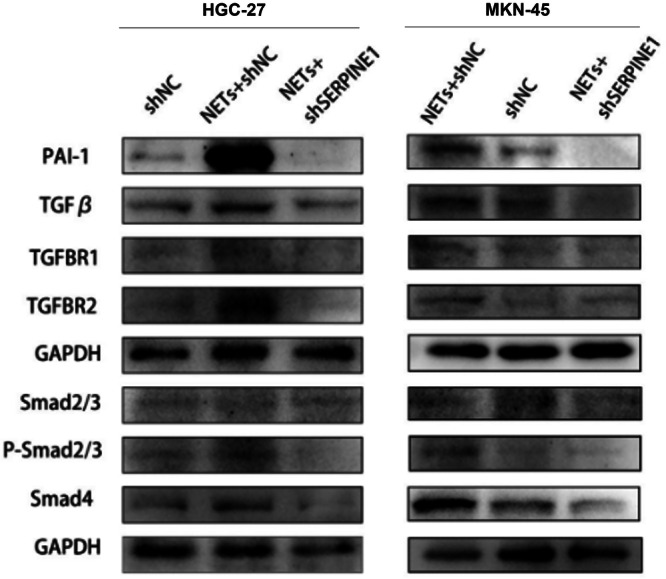
NETs activate the TGF‐β signalling pathway via PAI‐1. Western blot analysis of TGF‐β pathway components (TGF‐β1, TGF‐βR1, TGF‐βR2, phospho‐Smad2/3, total Smad2/3, and Smad4) in control and SERPINE‐1 knockdown HGC‐27 and MKN‐45 cells treated with NETs.

Western blot analysis revealed that NET treatment significantly increased the expression of TGF‐β1, TGF‐βR1, TGF‐βR2, phosphorylated Smad2/3 and Smad4 in both HGC‐27 and MKN‐45 cells (Figure 4). Importantly, SERPINE‐1 knockdown substantially reduced the NET‐induced upregulation of these TGF‐β pathway components. The total Smad2/3 levels remained relatively unchanged across all groups, suggesting that the observed effects were primarily due to changes in Smad2/3 phosphorylation rather than total protein levels.

These results indicate that NETs activate the TGF‐β signalling pathway in gastric cancer cells, and this activation is largely dependent on PAI‐1 expression.

### 3.5 SERPINE‐1 Knockdown Inhibits Neutrophil Extracellular Trap‐Mediated Liver Metastasis In Vivo

To validate the in vitro findings in an in vivo setting, a nude mouse model of gastric cancer liver metastasis was employed; HGC‐27 cells (control, NET + shNC or NET + shSERPINE‐1) were injected into the spleens of nude mice, and liver metastases were evaluated after 2 weeks.

**Figure 5:**
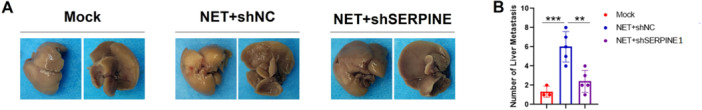
SERPINE‐1 knockdown inhibits NET‐mediated liver metastasis in vivo. (A) Representative images of liver metastases in nude mice injected with control HGC‐27 cells, NET‐treated HGC‐27 cells with shNC, or NET‐treated SERPINE‐1 knockdown HGC‐27 cells. (B) Quantification of liver metastases in each group. **p < 0.01, ***p < 0.001.

Strikingly, mice injected with NET‐treated HGC‐27 cells developed significantly more liver metastases compared with the control group (Figure 5). In contrast, mice injected with NET‐treated SERPINE‐1 knockdown cells showed a dramatic reduction in liver metastases, with numbers comparable with the control group. These results provide compelling in vivo evidence that NET‐induced, PAI‐1‐mediated EMT promotes gastric cancer metastasis.

## 4 Discussion

In this study, we have uncovered a novel mechanism by which NETs promote EMT and metastasis in gastric cancer through the upregulation of PAI‐1 and subsequent activation of the TGF‐β signalling pathway. Our findings provide important insights into the complex interplay between inflammatory components of the tumour microenvironment and cancer progression.

The overexpression of PAI‐1 in gastric cancer tissues and cell lines, as demonstrated by our immunohistochemistry (IHC) and western blot results, is consistent with previous studies linking PAI‐1 to poor prognosis in various cancers [15, 16, 17]. However, our work extends these findings by establishing a direct connection between NETs, PAI‐1 expression and EMT in gastric cancer. In this study, we found that PAI‐1 was highly expressed in gastric cancer. The IHC and western blot results showed that PAI‐1 expression was higher in gastric cancer tissues and cell lines.

The pro‐EMT effects of NETs observed in our study, including increased expression of mesenchymal markers (Vimentin and N‐cadherin) and decreased expression of the epithelial marker E‐cadherin, along with enhanced invasive and migratory capabilities, are in line with emerging evidence implicating NETs in cancer progression [18, 19]. NETs are reticulations released by neutrophils containing the DNA backbone and cytotoxic enzymes bound to them. DNase‐1 can disrupt the structure of NETs and reduce the formation and activity of NETs by degrading the DNA backbone in NETs [20]. After finding that NETs could promote EMT in gastric cancer, we used DNase‐1 to further validate this experimental result. Importantly, our finding that DNase‐1 treatment abrogates these effects underscores the critical role of NET structure integrity in mediating these pro‐tumorigenic functions.

PAI‐1 has previously been reported to promote the development of gastric cancer [11]. To further investigate the role of PAI‐1 versus NETs in gastric cancer, we used shRNA to decrease PAI‐1 expression. The results showed that NET‐induced EMT in gastric cancer was greatly attenuated after PAI‐1 knockdown. The reversal of NET‐induced EMT by SERPINE‐1 knockdown provides strong evidence for the central role of PAI‐1 in this process. This finding is particularly significant as it identifies PAI‐1 as a potential therapeutic target for preventing NET‐mediated gastric cancer progression. Moreover, the observed effects on sphere formation and cell proliferation suggest that PAI‐1 may also contribute to the acquisition of cancer stem cell‐like properties, a hallmark of aggressive tumours [21]. A recent study reported that PAI‐1 knockout exacerbated PAO1‐induced pneumonia‐associated injury and contributed to NET‐mediated pyroptosis and ferroptosis through PI3K/MAPK/AKT pathway activation [22]. This study also indicated that PAI‐1 was associated with NETs. To further investigate the NETs–PAI‐1 axis and how to regulate the EMT of gastric cancer, we detected the TGF‐β signalling pathway. The TGF‐β pathway is a well‐established inducer of EMT in various cancer types [23]. Our results showed that elevated PAI‐1 expression leads to activation of the TGF‐β signalling pathway. Our mechanistic studies revealing NET‐induced activation of the TGF‐β signalling pathway via PAI‐1 provide a plausible explanation for the observed pro‐EMT effects, and our results suggest that NETs may exploit this pathway through PAI‐1 upregulation to promote gastric cancer progression. This finding opens up new possibilities for therapeutic interventions targeting the NET–PAI‐1–TGF‐β axis.

To further validate our conclusion, the in vivo validation of our findings using a nude mouse model of gastric cancer liver metastasis provides compelling evidence for the clinical relevance of our observations. The dramatic reduction in liver metastases following SERPINE‐1 knockdown in NET‐treated cells highlights the potential of targeting PAI‐1 as a strategy to prevent gastric cancer metastasis.

While our study provides significant insights into the role of NETs in gastric cancer progression, it also raises several important questions for future research. For instance, the precise mechanisms by which NETs upregulate PAI‐1 expression in gastric cancer cells remain to be elucidated. Additionally, investigating the potential bidirectional relationship between NETs and PAI‐1, where PAI‐1 may influence NET formation or stability, could provide a more comprehensive understanding of this pathway.

Furthermore, our findings suggest potential therapeutic strategies for gastric cancer. Targeting NETs through DNase‐1 treatment or inhibiting PAI‐1 could be promising approaches to prevent EMT and metastasis. However, careful consideration must be given to potential side effects, as both NETs and PAI‐1 play important physiological roles in inflammation and haemostasis [24, 25]. It is also worth noting that while our study focused on gastric cancer, the NET–PAI‐1–TGF‐β axis may be relevant in other cancer types. Future studies exploring this pathway in different malignancies could reveal common mechanisms of cancer progression and potentially lead to broadly applicable therapeutic strategies.

## 5 Conclusion

Our study reveals a novel mechanism by which NETs promote EMT and metastasis in gastric cancer through PAI‐1 upregulation and subsequent TGF‐β signalling pathway activation. Furthermore, in vivo studies showed that SERPINE‐1 knockdown significantly reduces NET‐mediated liver metastasis in a mouse model of gastric cancer. These findings not only advance our understanding of the complex interactions between inflammatory components and cancer progression but also identify potential therapeutic targets for preventing gastric cancer metastasis. Future research should focus on translating these insights into clinical applications, potentially through the development of NET‐targeting or PAI‐1 inhibition strategies, and exploring the broader implications of the NET–PAI‐1–TGF‐β axis in other cancer types to develop more comprehensive cancer treatment approaches.

## Author Contributions

Zhen Ma and Xiaolin Li conceived of the study. Shifeng Yang, Hao Yang and Ange Zhang participated in its design and data analysis and statistics. Nana Li and Xiaoming Zou helped to draft the manuscript. All authors read and approved the final manuscript.

## Ethics Statement

This study was conducted in accordance with the Declaration of Helsinki and approved by the Ethics Committee of The Second Affiliated Hospital of Harbin Medical University (Approval Number: YJSKY2022‐227). All laboratory operations on animals followed the Guidelines for the Care and Use of Laboratory Animals.

## Consent

Written informed consent was obtained from all participants.

## Conflicts of Interest

The authors declare no conflicts of interest.

## Data Availability

All data generated or analysed during this study are included in this article. Further enquiries can be directed to the corresponding author.
